# Limited Genetic Diversity of Hepatitis B Virus in the General Population of the Offin River Valley in Ghana

**DOI:** 10.1371/journal.pone.0156864

**Published:** 2016-06-06

**Authors:** Kobina Assan Ampah, Carlos Augusto Pinho-Nascimento, Sarah Kerber, Prince Asare, Daniel De-Graft, Frank Adu-Nti, Izabel C. N. P. Paixão, Christian Niel, Dorothy Yeboah-Manu, Gerd Pluschke, Katharina Röltgen

**Affiliations:** 1 Swiss Tropical and Public Health Institute, Molecular Immunology, Basel, Switzerland; 2 University of Basel, Basel, Switzerland; 3 Noguchi Memorial Institute for Medical Research, University of Ghana, Legon, Ghana; 4 Laboratory of Molecular Virology, Biology Institute, Fluminense Federal University, Niterói, Brazil; 5 Radford University College, Accra, Ghana; 6 Laboratory of Molecular Virology, Oswaldo Cruz Institute, Fiocruz, Rio de Janeiro, Brazil; Defence Research Laboratory, INDIA

## Abstract

Hepatitis B virus (HBV) infections account for approximately 780,000 deaths per year, most of which occur in the developing world. Co-infection with HBV and hepatitis delta virus (HDV) may lead to the most severe form of viral hepatitis. In Ghana, knowledge on the prevalence of HBV and HDV in the general population is scanty and the few genetic analyses of the prevailing HBV genotypes are dating back more than a decade. In the present study, 1,323 serum samples from individuals living in a rural area (Offin river valley) of Ghana were analyzed for the presence of the hepatitis B surface antigen (HBsAg). Positive sera were subsequently tested for the presence of anti-HDV antibodies. A total of 107 (8%) sera were HBsAg positive with an 8.4% prevalence of anti-HDV antibodies among the HBsAg positives. Phylogenetic analysis based on HBV pre-S/S sequences, attributed all 52 typable samples to genotype E. All belonged to serotype *ayw4*. While 19 sequences clustered with those from a number of African countries, the other 33 formed a separate cluster distinguished by an intergroup mean distance of 1.5% from the pan-African HBV/E cluster. Successful implementation of HBV vaccination in the region was reflected by the low HBsAg carrier rate of 1.8% among children ≤11 years.

## Introduction

Despite the availability of effective hepatitis B virus (HBV) vaccines, the global burden of hepatitis B remains high, with an estimated 240 million chronically infected individuals and about 780,000 deaths from cirrhosis and hepatocellular carcinoma each year [1]. Transmission of HBV can occur through diverse routes including perinatal, sexual and household contact, or by unsafe injections. The HBV genome is a circular, partially double-stranded DNA, approximately 3.2 Kb in length, which contains four partially overlapping open reading frames, encoding the polymerase (P), the surface proteins (pre-S1/pre-S2/S), the core antigen and the soluble antigen ‘e’ (preC/C), and the regulatory protein (X) [2]. To date, HBV isolates have been classified into eight confirmed (A to H) [3–7] and two tentative (I and J) [8, 9] genotypes, based on a divergence of >7.5% in the whole genome, or >4% in the S gene sequence. In recent years it has become increasingly evident that a distinct global geographical distribution of HBV genotypes is a major factor responsible for differences observed in clinical manifestations and response to antiviral treatment and vaccination [10–12], emphasizing the importance of genotyping studies to identify the locally prevailing genotypes.

While hepatitis B is hyper-endemic in sub-Saharan Africa, underreporting due to limited access to healthcare and lack of knowledge on the infection hinder precise accounts on the actual HBV burden, particularly in remote areas [13]. Previous studies have shown that HBV genotype E (HBV/E) is by far the most prevalent in West and Central Africa [14–17], spreading in a vast crescent with a span from Senegal to Namibia. The other two dominating genotypes circulating in Africa, are found mainly in Southern, Eastern and Central Africa (HBV/A) [13], and in Northern Africa (HBV/D) [18–20]. A recent emergence of genotype E, within the last 130 years, has been proposed, based on the fact that this genotype is restricted to the African continent and shows the lowest diversity, as compared to other genotypes [14, 21, 22]. However, the origin and evolutionary history of HBV/E remain unclear. While recent studies in Ghana have investigated hepatitis B surface antigen (HBsAg) seroprevalence rates within specific groups, such as HIV patients (13–17% [23, 24]), pregnant women (11–13% [25–27]), blood donors (7.5–15% [28–31]), or prison inmates (17–25.5% [32, 33]), knowledge of HBV prevalence in the general population is limited. Although it has been reported that HBV/E is the prevailing genotype in historical specimens from Ghana [34, 35], detailed information on the current population structure of HBV in the country is lacking.

An estimated 15–20 million individuals worldwide are co-infected with HBV and hepatitis delta virus (HDV), representing the most severe form of chronic hepatitis [36, 37]. HDV is a defective virus requiring HBsAg to survive, and is principally transmitted by the parenteral route. Except for a recent study reporting an 11.3% seroprevalence of HDV infection in a small number of patients with HBV-related liver diseases in Accra [38], data on the prevalence of HDV in Ghana are lacking.

The objective of the present study was to investigate the prevalence and genomic diversity of HBV in the general population of the Offin river valley in Ghana.

## Materials and Methods

### Ethics statement

Ethical approval for the study was obtained from the institutional review board of the Noguchi Memorial Institute for Medical Research (Federal-wide Assurance number FWA00001824). Written informed consent was provided by all study participants or, in the case of children, by their parents or guardians.

### Design of the study

**Fig 1:**
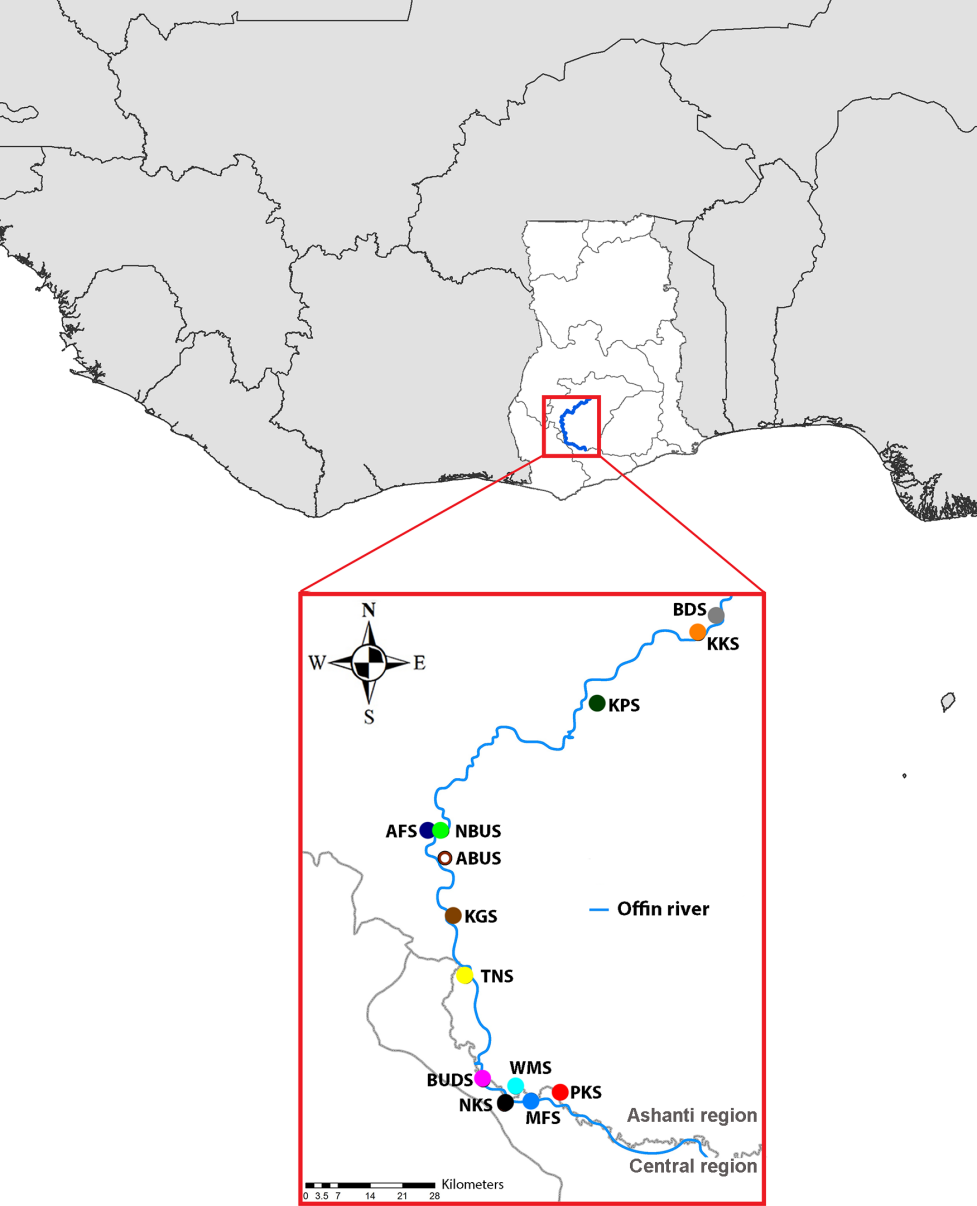
Study area. Map of Ghana with the Offin river valley and surrounding countries. The 13 study communities along the Offin River are indicated as multicolored dots: Bedomase (BDS); Krakrom (KKS); Kapro (KPS); Akomfore (AFS); Ntobroso (NBUS); Achiase (ABUS); Keniago (KGS); Tontonkrom (TNS); Dominase (BUDS); Wromanso (WMS); Nkotumso (NKS); Mfantseman (MFS); Pokukrom (PKS). The grey lines in the Ghana map indicate the borders of the Ghanaian regions. The background maps were created using the *ArcMap* program in *ArcGIS* v.10.0 and were modified with Adobe Photoshop CS6.

The Offin river runs through two (Ashanti and Central) of the ten Ghanaian regions (Fig 1), covering a total of 11 health districts. In a preceding study, we investigated the epidemiology of the tropical skin disease Buruli ulcer in selected communities of the Offin river valley of Ghana by conducting an exhaustive household survey in 2013 [39]. Based on the demographic information collated for 20,390 residents of 13 communities located in seven health districts (Table 1) spread along the Offin river (Fig 1), we grouped the population of each community by age and randomly selected from all of the age groups a total of 1,560 residents (120 per community). Of these 1,560 individuals, 1,352 consented to donate blood for sero-epidemiological studies of multiple pathogens [40].

**Table 1:** Characteristics of the study population and HBsAg and anti-HDV antibody seroprevalences.

Variables	Community*													Total
	ABUS	AFS	BDS	BUDS	KPS	KGS	KKS	MFS	NKS	NBUS	PKS	TNS	WMS	—
Health districts‡	AM	AM	SS	UDW	AN	AW	SS	UDE	UDW	AM	UDE	AW	AC	—
**Community Infrastructure**														
Road network§	C2	C3	C2	C1	C1	C2	C3	C3	C1	C2	C3	C2	C3	—
Social infrastructure#	SRM	SR	SRM	SRMH	SR	SRMH	-	SRM	SRMH	SRMH	SRMH	SRMH	SR	—
**Surveyed Population**														
Total inhabitants, n	1,900	1,016	1,688	2,802	692	3,350	111	303	2,518	1,949	900	2,945	216	20,390
Recent travel history, n (%)	518 (27.3)	350 (34.4)	546 (32.3)	1,360 (48.5)	192 (27.7)	1,156 (34.5)	67 (60.4)	78 (25.7)	1,120 (44.5)	758 (38.9)	409 (45.4)	1,117 (37.9)	141 (65.3)	7,812 (38.3)
Recent contact with major cities, n (%)	354 (18.6)	150 (14.8)	357 (21.1)	1,036 (37.0)	96 (13.9)	705 (21.0)	11 (9.9)	10 (3.3)	462 (18.3)	446 (22.9)	281 (31.2)	662 (22.5)	89 (41.2)	4,659 (22.8)
**Study Participants**														
Total, n	117	113	112	91	111	107	71	114	80	123	116	94	74	1,323
Females, n (%)	64 (54.7)	64 (56.6)	68 (60.7)	48 (52.7)	62 (55.9)	66 (61.7)	40 (56.3)	65 (57.0)	48 (60.0)	77 (62.6)	82 (70.7)	49 (52.1)	48 (64.9)	781 (59.0)
HBsAg sero-prevalence, n (%)	3 (2.6)	12 (10.6)	3 (2.7)	9 (9.9)	11 (9.9)	2 (1.9)	7 (9.9)	6 (5.3)	10 (12.5)	11 (8.9)	10 (8.6)	14 (14.9)	9 (12.2)	107 (8.1)
Anti-HDV antibody sero-prevalence among HBsAg pos, n (%)	1 (33.3)	0 (0)	0 (0)	1 (11.1)	0 (0)	0 (0)	0 (0)	0 (0)	3 (30)	0 (0)	0 (0)	3 (21)	1 (11.1)	9 (8.4)

* ABUS = Achiase; AFS = Akomfore; BDS = Bedomase; BUDS = Dominase; KPS = Kapro; KGS = Keniago; KKS = Krakrom; MFS = Mfantseman; NKS = Nkotumso; NBUS = Ntobroso; PKS = Pokukrom; TNS = Tontonkrom; WMS = Wromanso

‡ AM = Atwima Mponua district; SS = Sekyere south district; UDW = Upper Denkyira West district; AN = Atwima Nwabiagya district; AW = Amansie West district; UDE = Upper Denkyira East district; AC = Amansie Central district

§ C1 = asphalt road surface, major road connecting one district capital to another with relatively high traffic volume; C2 = gravel road surface, major road connecting one town to another with relatively low traffic volume; C3 = gravel road surface, minor road connecting one town to another with cars sparingly plying the road

# S = school; R = religious facilities; M = central market; H = health center

In the present study, we analyzed 1,323 of the 1,352 serum samples retrospectively for the presence of HBV and HDV markers. The distribution of study participants by age group and community of residence is shown in S1 Fig. Moreover, we re-evaluated general information on the communities, as well as study questionnaires administered in 2013 to 20,390 inhabitants of the 13 study communities, to extract demographic information relevant for the study of hepatitis in this region, including road networks, social and health system infrastructure and travel habits.

### Serological analysis

A total of 1,323 retrospective blood serum samples stored at -80°C were analyzed in the present study.

#### Detection of HBsAg

Serum samples were screened for the presence of HBsAg by immunochromatography (Advanced Quality ONE STEP HBsAg Test Strip, Intec Products Inc., China) according to the manufacturer’s instructions. Briefly, we applied 100 μl of serum onto the test strips and recorded the presence or absence of a red band on the nitrocellulose strip after 15 minutes. In order to validate the performance of this rapid test, we analyzed 88 serum samples in parallel by ELISA using the HBsAg BioAssay ELISA Kit (US Biological, Salem, USA) in strict accordance to the manufacturer’s instructions. The same results were obtained in both tests, with three samples yielding a positive result.

#### Detection of anti-HDV antibodies

The HDV antigen (HDAg) can elicit a specific antibody response in infected individuals. We analyzed all HBsAg-positive serum samples for the presence of anti-HDV antibodies by ELISA (ETI-AB-DELTAK-2, DiaSorin, Italy) according to the manufacturer’s instructions.

### Nucleic acid extraction, HBV PCR amplification and DNA sequencing

Total nucleic acids were extracted from 200 μl of HBsAg-positive serum samples using the High Pure Viral Nucleic Acid Kit (Roche, Mannheim, Germany) according to the manufacturer's instructions. Nucleic acids were re-suspended in 50 μl of TE buffer and 3 μl were applied in the PCR assays.

Amplification of the HBV pre-S/S region was attempted by a semi-nested PCR assay, in which samples negative in the first round, were subjected to a second round of PCR. In both reactions, PCR amplification of extracted DNA was performed with 2.5 units of FirePolTaq-Polymerase (Solis BioDyne, Tartu, Estonia), FirePol buffer, 2 mM MgCl_2_ and 0.4 mM dNTPs with 1.8 μl of 10 μM forward and reverse primers each in a total volume of 30 μl. While primer pairs PS1 (5’-CCATATTCTTGGGAACAAGA-3’) and P3 (5’-AAAGCCCAAAAGACCCACAA-3’) were applied in the first PCR round to generate a product of 1,405 bp, P3 was replaced by S2 (5’-GGGTTTAAATGTATACCCAAAA-3’) in the second round, amplifying a product of 1,227 bp. PCR reactions were carried out in a Gene Amp PCR System 9700 PCR machine (Applied Biosystems) and thermal conditions for PCR amplifications included an initial denaturation step at 94°C for 5 min followed by 32 cycles at 94°C for 30 s, 57°C for 30 s and 72°C for 1 min and a final extension step, 72°C for 10 min. PCR products were analyzed on 1% agarose gels. Nucleotide sequencing was accomplished using primers PS1, PS8 (5’-TTCCTGAACTGGAGCCACCA-3’), PS4 (5’-ACACTCATCCTCAGGCCATGCAGTG-3’), S2, S4 (5’-TGCTGCTATGCCTCATCTTCT-3’) and P3 for the larger sequence, and PS1, PS8, PS4 and S2 for the smaller sequence. We also amplified a smaller fragment spanning the S gene by applying the same PCR procedure using primers PS1a (5’-GGAAAACATCACATCAGGAT-3’) and P3 for the first round of PCR and primers PS1b (5’-AAAATTCGCAGTCCCCAACC-3’) and P3 for the second round. In this case, the same primers were used for DNA sequencing. PCR products were purified using the Nucleo Spin Extract II Kit (Macherey-Nagel, Düren, Germany) and sequenced at Macrogen Inc., Europe (Amsterdam, the Netherlands).

### Statistical analysis

All statistical analyses were carried out with GraphPad Prism version 6.0 (GraphPad Software, San Diego, CA) and Stata 12 (Statacorp 2011 statistical software Release 12. College Station, TX: StataCorp LP). The associations between categorical variables were assessed using Pearson’s Chi square test or Fisher’s exact test. The level of statistical significance was set P<0.05.

### Phylogenetic analysis and inference of serotypes

Genotyping of HBV was performed by analyzing a partial sequence of the genome—the pre-*S/S* region—consistent with genotyping of the full genomic sequence. Nucleotide sequences of the pre-*S/S* or *S* genes were aligned using the ABI Prism AutoAssembler, version 1.4.0 (Perkin-Elmer, Waltham, MA). Maximum-likelihood phylogenetic analysis was performed using the Kimura 2-parameter model [41] of MEGA version 6.0 [42]. Previously published mutations in the *S* region associated with “escape” or with diminished antibody binding were predicted using the Geno2pheno[HBV] online tool at http://hbv.geno2pheno.org/index.php. The Geno2pheno[HBV] tool was also used to predict drug resistance mutations in the part of the reverse transcriptase open reading frame that overlaps with that of *S*. Genetic distances were estimated using MEGA 6.0 tools [42].

HBV serotypes were predicted based on the amino acids present at either three or five known positions (122, 160, 127, 159 and 140) within the *S* gene [43] using the web-based HBV Serotyper Tool that can be accessed at http://hvdr.bioinf.wits.ac.za/SmallGenomeTools [44].

### DNA sequence accession numbers

The HBV/E sequences determined in this work have been deposited in the DDBJ/GenBank/EMBL database (accession numbers KU522251-KU522302).

## Results

### Demographic characteristics of the study population and study participants

In order to gain insight into the burden of hepatitis B and D infection in the general population of the Offin river valley in Ghana, we analyzed serum samples from a representative proportion of individuals living in 13 selected communities located up-, mid- and downstream along the Offin river (Fig 1). Of the total of 20,390 residents of these communities, 1,323 individuals (from 71 to 123 inhabitants per community) aged between 1 and 90 years (mean age 25.3 ± 19.5 years, median 19 years) participated in the study (S1 Fig), with 59% (n = 781) being females and 41% (n = 542) being males (Table 1). The 13 communities belonged to seven health districts, all of which reported to have introduced the pentavalent diphtheria, tetanus, pertussis, *Haemophilus influenzae* type B, hepatitis B (DPTHH) vaccine in 2002 following the WHO Expanded Program on Immunization (EPI).

As listed in Table 1, the characteristics of the 13 selected study communities varied, ranging from small settlements located within farmlands and lacking basic social amenities and a good road network, like Krakrom, to larger communities, like Dominase, which in addition to having all the social amenities, is connected to the district capital by a good road network. Almost 40% (7,812/20,390) of the general population reported to have travelled out of their communities in the past three months with the majority of those (n = 4,659) reporting to have resided in a metropolitan town for at least one day. While 80% (16,262/20,390) of the inhabitants were Akans, the other 20% were composed of Ewe, Mole, Ga/Ada, Guan, Gruma, Grusi, Mande, Mamprusi, Kussasi and others [39]. There were also 37 (0.18%) nationals of other West African countries, namely Benin (n = 2), Burkina Faso (n = 7), Côte d'Ivoire (n = 4), Togo (n = 4), Niger (n = 12) and Nigeria (n = 8).

### Prevalence of HBsAg and anti-HDV antibodies in the Offin River Valley population

**Fig 2:**
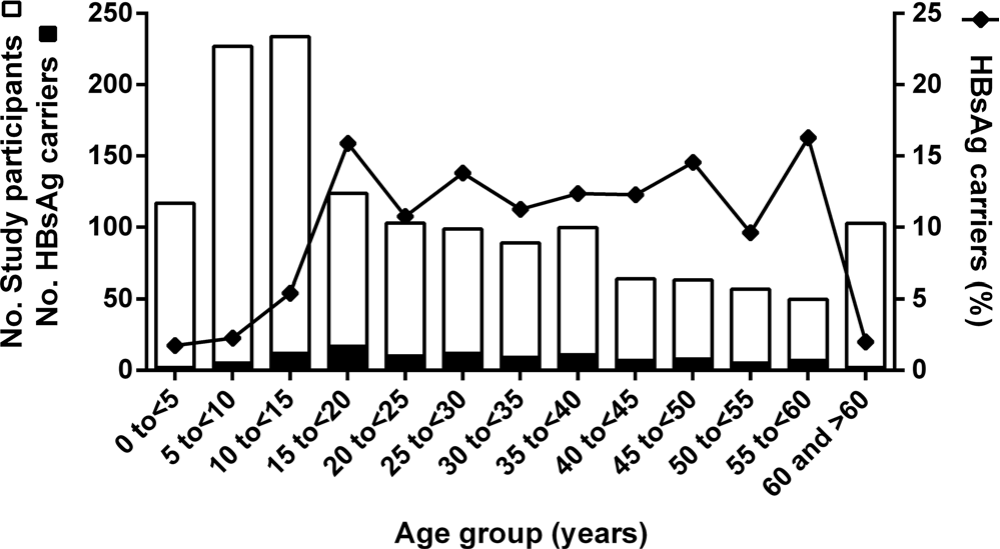
Age distribution and percentage of HBsAg carriers. A stacked graph of the total number of study participants for each age group (white bar) and of the corresponding number of HBsAg carriers (black bar) (left y-axis) is shown. The percentage of HBsAg carriers for each age group (right y-axis) is indicated by squares.

One hundred and seven (8.1%) of the 1,323 serum samples tested were HBsAg positive, with a nearly equal gender distribution of 7.7% (n = 60) in females and 8.7% (n = 47) in males (p = 0.516). There was no significant association between ethnic groups and positivity for HBsAg (p = 0.609), but a high variation in prevalence (1.9% to 14.9%) between communities, which was however not associated with the quality of road or social infrastructure of the community (Table 1). The age distribution of HBsAg positive individuals in relation to the total number of study participants is depicted in Fig 2. HBsAg was detected in a significantly (p < 0.001) higher proportion of individuals aged above 11 years (11.1%; 99/890) than in children aged 11 years or less (1.8%; 8/433). Prevalence of HBsAg-positivity was low (2%; 2/101) in the age group ≥60 years.

Nine (8.4%) of the 107 HBsAg carriers were tested positive for anti-HDV antibodies. All nine positive sera (ABUS036, BUDS073, NKS076, NKS089, NKS100, TNS007, TNS058, TNS088 and WMS021) were from residents of five study communities (Table 1) located mid- and downstream along the Offin river (Fig 1).

### Phylogenetic analysis of HBV isolates circulating in the Offin river valley

HBV DNA could be amplified from 66/107 (62%) samples positive for HBsAg. Phylogenetic analysis was performed with pre-S/S sequences obtained from 52 of the 66 PCR products. The remaining 14 sequences showed high similarity to the other sequences obtained, but each of them contained several ambiguous nucleotide positions (suggesting mixed infections with more than one genetic HBV variant), and the sequences were therefore not included in the phylogenetic analysis.

**Fig 3:**
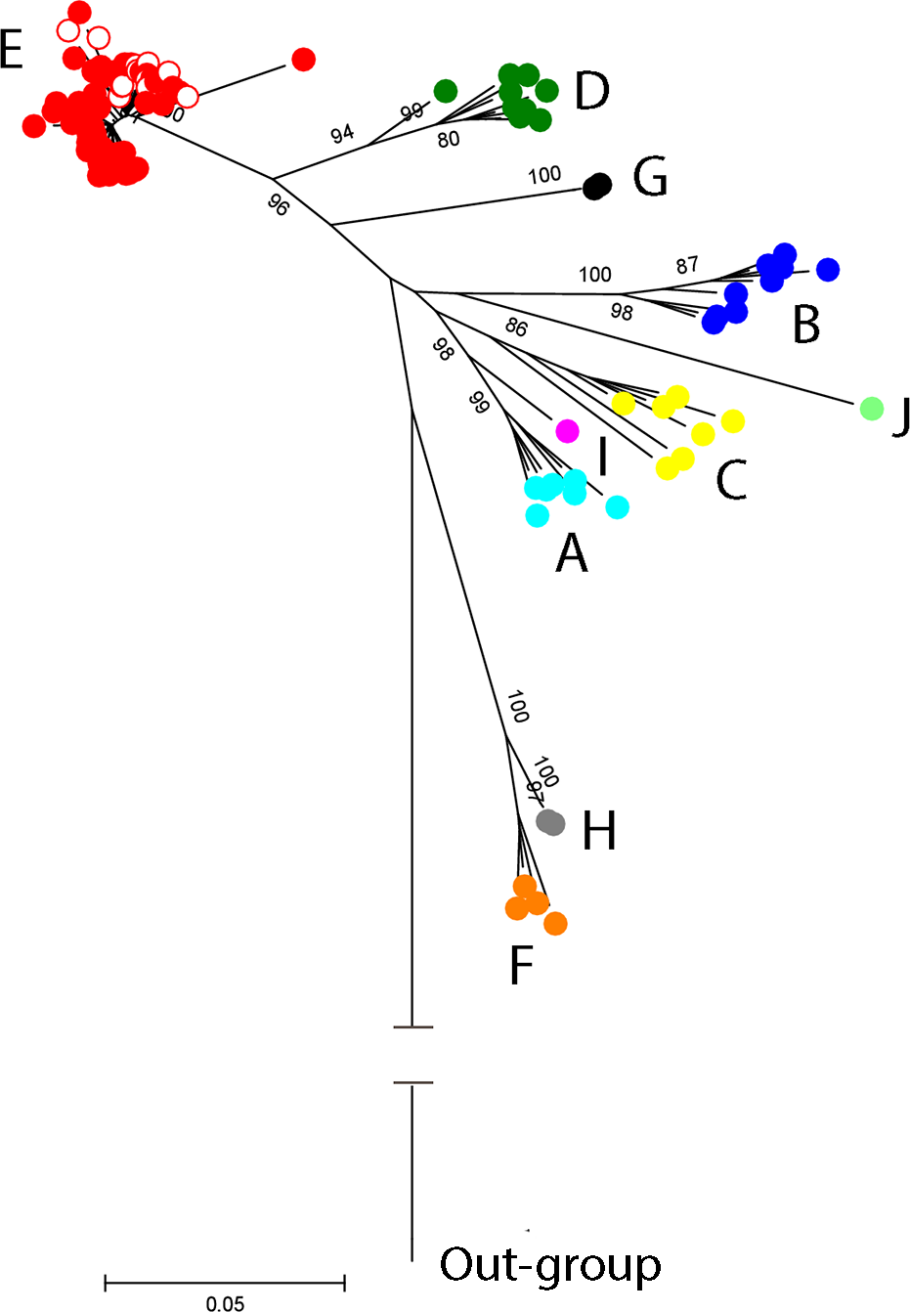
Phylogenetic reconstruction of world-wide and Offin river valley HBV sequences based on the pre-S/S region. A maximum-likelihood phylogenetic tree of HBV pre-S/S sequences obtained in this study (red dots) together with publically available sequences covering all HBV genotypes (multicolored dots) was constructed with 1000 bootstrap replicates using the Kimura 2-parameter +G +I model [41] contained in MEGA6 [42]. The tree is drawn to scale, with branch lengths corresponding to the number of substitutions per site. Reference sequences retrieved from GenBank are for genotypes A (AY233278, HE576988, AM184126, AM180623, FJ692609, GQ331047, FN545833), B (AB073842, AB073836, AB033555, AB100695, AB219427, DQ463789, EF473977, AP011093, GQ358145), C (AF223954, AB033556, X75656, AB048704, AB241109, AP011102, AP011104), D (JF754615, EU594428, AY233291, KF192838, GQ205378, AB493846, FJ904430, FN594771, JN664919), E (circles without fill: AB205192, AB106564, DQ060830, AB201290, AB205188, AB091255, AB091256, HM363611, FN594765, AB205191, AB205190), F (AF223964, X69798, AB036914, DQ823087), G (AB056513, EF634480), H (EU498228, EF157291), I (FJ023660), and J (AB486012). The sequence of a Woolly monkey HBV was included as an out-group.

The 52 sequences (obtained from residents of 12 of the 13 study communities) were compared with representative sequences of the HBV isolates from genotypes A-J retrieved from GenBank (see accession numbers in the legend of Fig 3). The resulting phylogenetic reconstruction was rooted by including the sequence of a virus isolate from a Woolly monkey (WMHBV) as an out-group (GenBank accession number AY226578). All Offin river valley sequences clustered with those of HBV/E strains from Ghana and other countries included in the alignment (Fig 3).

**Fig 4:**
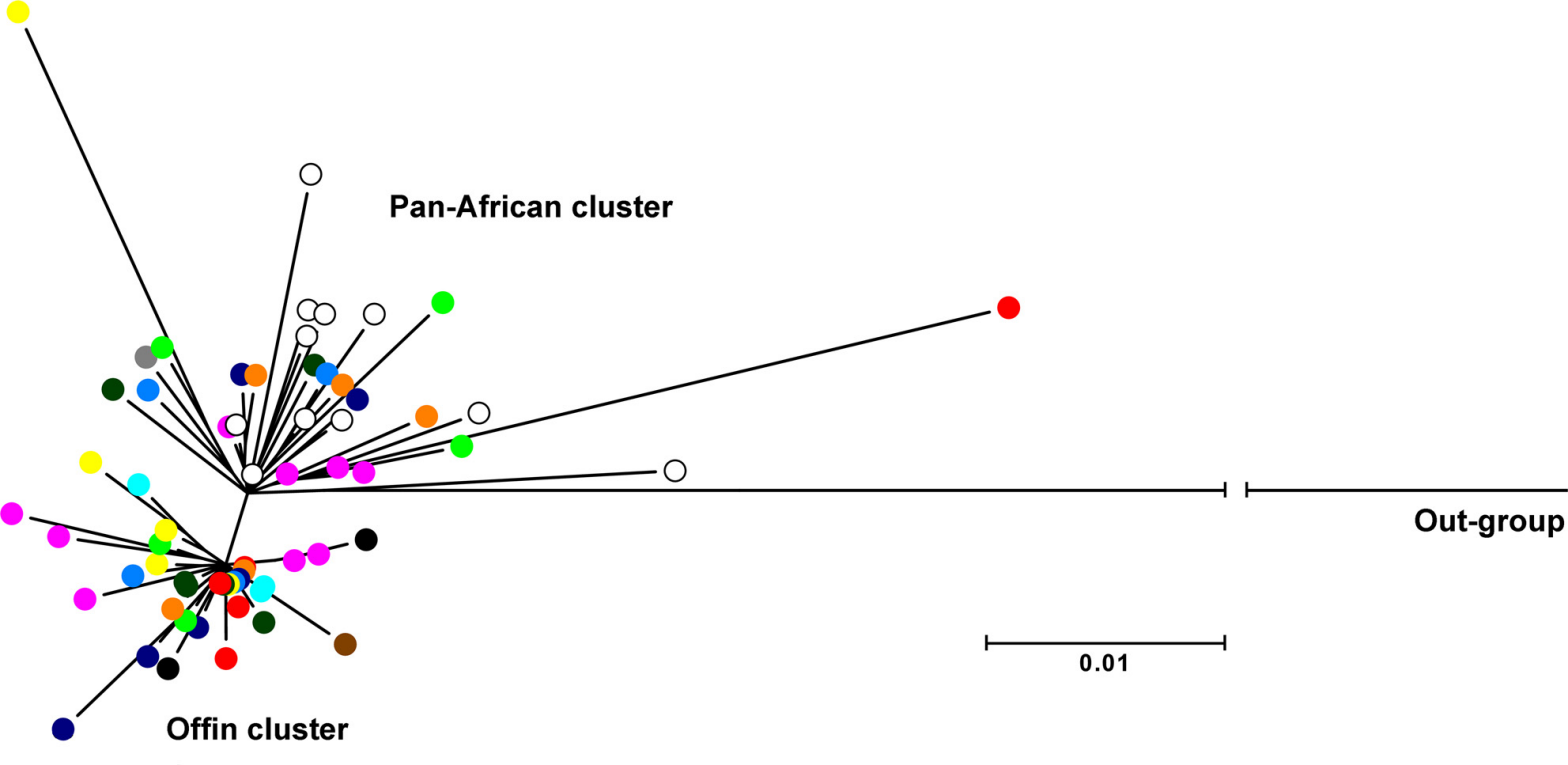
Phylogenetic reconstruction of HBV/E sequences. A maximum-likelihood phylogenetic tree based on the pre-S/S gene region sequence (1,200 bp) was constructed using the Kimura 2-parameter +G model [41] embedded in MEGA6 [42]. While HBV sequences obtained in this study are indicated as multicolored dots (according to village of residence), HBV genotype E sequences retrieved from GenBank are depicted as empty circles (accession numbers (origin): AB205192 (Ghana), AB106564 (Ghana), DQ060830 (Madagascar), AB201290 (Benin), AB205188 (Ghana), AB091255 (Côte d'Ivoire) AB091256 (Côte d'Ivoire), HM363611 (Nigeria), FN594765 (Niger), AB205191 (Ghana), AB205190 (Ghana)). The sequence of the genotype D AY233291 from South Africa was included as an out-group.

A more detailed resolution of the population structure of HBV from the Offin river valley was achieved by aligning the sequences exclusively with other HBV/E sequences. The overall mean genetic distance among the 52 sequences of this study was 1.2% based on the pre-S/S region, and 0.6% when only the S region was analyzed. Interestingly, maximum likelihood reconstruction, based on the complete pre-S/S region, revealed the presence of two separate genetic clusters, with an intergroup mean distance of 1.5%. While 19 sequences grouped with HBV/E strains from Ghana, Madagascar, Benin, Côte d'Ivoire, Nigeria, and Niger (pan-African cluster), the 33 others formed a separate cluster that did not contain any sequence other than those from the Offin river valley (Fig 4). Representatives of the Offin cluster were from 11 of the 13 study communities, while sequences belonging to the pan-African cluster originated from nine communities (S1 Table). However, no significant association was found between sequence origin and affiliation to one of the two different clusters (p = 0.589). The mean genetic distance of the 19 sequences attributed to the pan-African cluster was 1.6%, compared with 0.7% among the 33 belonging to the Offin cluster. Characteristics of all individuals for which sequence information is available are listed in S2 Table.

### Detection of relevant HBV signature motifs, deletions and other mutations in the pre-S/S region

The deduced amino-acid sequences of the pre-S/S region were highly conserved among the 52 HBV isolates analyzed in this study. All of them had a single amino acid deletion at the N-terminus of the pre-S1 region (residue Met^12^ in sequences of other genotypes), leading to a pre-S1 region of 118 amino acids in length, a feature characteristic for HBV/E strains [45]. Moreover, all sequences showed the genotype E pre-S1 amino acid signature motif Leu^3^SerTrpThrValProLeuGluTrp^11^. Amino acid residue Met^83^, that has been reported to introduce a new translational start codon in the pre-S1 region [45], was present in all but one (NBUS067) sequence. The emergence and selection of new pre-S variants is a common event in chronically HBV infected patients. Genetic defects are usually due to in-frame deletions of different sizes in the carboxy terminus of the pre-S1 region, or are caused by substitutions at the start codon of the pre-S2 region with complete abolishment of M protein synthesis [46]. Here, deletions of 27 and 25 amino acids (sample PKS034) and of six amino acids (PKS077) were detected in the pre-S region. Moreover, substitutions leading to the loss of the pre-S2 start codon (Met^119^) were detected in three sequences (NBUS058, NKS010 and WMS009).

The S region is particularly conserved. It has been reported that the majority of the HBV/E isolates from South-West Africa contain an Ile^57^ residue, while those from North-West Africa show a Thr^57^ residue [45]. In this study, 51 Ghanaian sequences had a Thr residue, while only one (TNS014), belonging to the pan-African cluster, had an Ile at that position.

Neutralizing antibodies induced by immunization against HBV are mainly directed against conformational epitopes of the major antigenic ‘a’ determinant, spanning amino acids 124–147 of the S region [47]. A number of amino acid substitutions within this region, mainly between positions 137 and 147, have been described as vaccine or immune escape mutants [48]. In two of the HBV sequences obtained in this study (MFS048 and TNS091), we detected the mutation sS143L, previously identified in an HBsAg escape mutant [49]. No known mutations in the S-overlapping reverse transcriptase region, relevant for phenotypic resistance to five antiretroviral drugs (Lamivudine, Adefovir, Entecavir, Tenofovir and Telbivudine) were found.

Two nonsense mutations of the S gene—one at position Leu^216^ (sLeu216*) affecting six HBV sequences (PKS034, TNS014, WMS009, BUDS073, TNS055 and TNS065), and the other at position Trp^182^ (sTrp182*) found in sequence BUDS005—were detected, resulting in a C-terminal truncation of the S protein in these strains. All deletions and substitutions described above are shown in Table 2.

**Table 2:** Comparison of deduced amino acids of sequences obtained in this study and HBV/E consensus.

	Pre-S region				S region									
	Met^83^	Gly^71^-Asn^97^, Arg^112^-Arg^136^	Val^135^-Phe^140^	Met^119^	Thr^57^	Gln^101^	Ile^110^	Ser^114^	Leu^127^	Ser^140^	Ser^143^	Gly^159^	Trp^182^	Leu^216^
**AFS097**	.	.	.	.	.	.	.	.	Pro	.	.	.	.	.
**AFS115**	.	.	.	.	.	.	.	.	Pro	.	.	.	.	.
**BUDS005**	.	.	.	.	.	His	.	.	.	.	.	.	*	.
**BUDS006**	.	.	.	.	.	.	Leu	Ala	.	.	.	.	.	.
**BUDS073**	.	.	.	.	.	.	.	.	.	.	.	.	.	*
**KKS062**	.	.	.	.	.	.	.	Pro	.	.	.	.	.	.
**KKS064**	.	.	.	.	.	.	.	.	Ile	.	.	.	.	.
**KPS088**	.	.	.	.	.	.	.	.	.	.	.	Ala	.	.
**MFS048**	.	.	.	.	.	.	.	.	.	.	Leu	.	.	.
**NBUS058**	.	.	.	Thr	.	.	.	.	.	.	.	.	.	.
**NBUS062**	.	.	.	.	.	.	.	.	.	.	.	.	.	.
**NBUS067**	Lys	.	.	.	.	.	.	.	.	.	.	.	.	.
**NBUS070**	.	.	.	.	.	.	.	Thr	Pro	.	.	.	.	.
**NBUS078**	.	.	.	.	.	His	.	.	.	.	.	.	.	.
**NKS010**	.	.	.	Ile	.	.	.	.	.	.	.	.	.	.
**PKS034**	.	——	.	.	.	.	.	.	.	.	.	Ala	.	*
**PKS077**	.	.	——	.	.	.	.	.	.	.	.	.	.	.
**TNS014**	.	.	.	.	Ile	.	.	.	.	Leu	.	.	.	*
**TNS055**	.	.	.	.	.	.	.	.	.	.	.	.	.	*
**TNS065**	.	.	.	.	.	.	.	.	.	.	.	.	.	*
**TNS091**	.	.	.	.	.	.	.	.	.	.	Leu	.	.	.
**WMS009**	.	.	.	Thr	.	.	.	.	.	.	.	.	.	*

Only relevant mutations and deletions described in the text are shown.

* nonsense mutation

### Serotype distribution of HBV in the Offin river valley

According to the previously described amino acid sequence algorithms [43], R^122^, K^160^, L/I^127^ (found in 94% of the sequences) and R^122^, K^160^, P^127^, G^159^, S^140^ (detected in the remaining 6%), all the 52 HBV sequences of this study were classified as HBV serotype *ayw4*.

## Discussion

Analysis of sera collected from inhabitants of 13 communities located in the Offin river basin of Ghana by a rapid strip assay revealed a high (>8%) HBsAg seroprevalence in the rural population under study. Since it is known that the sensitivity of rapid strip assays may be lower than that of more complex enzyme immunoassays [50], we validated the performance of the rapid test used by analyzing a subset of samples in parallel by an ELISA. Both assays yielded identical results for the 88 samples tested. Antibodies to HDV were found in the serum of more than 8% of the 107 HBsAg carriers. This frequency of co-infection is similar to that observed in a recent study conducted in Accra, where a HDV seroprevalence of 11.3% was found among 53 patients with HBV-related liver disease [38]. HBV can be transmitted horizontally by exposure to infected blood and various other body fluids or vertically by spread from mother to child at birth, with the majority of the children infected before the age of 6 months becoming chronic carriers [1]. In our study population we observed a significantly lower HBsAg carrier rate (1.8%) among children ≤11 years than in the older population (11.1%), coinciding with the 11 year period since when Ghana has introduced HBV vaccination with the pentavalent DPTHH vaccine. These data tend to confirm that HBV vaccination is effectively implemented in the Offin river valley. In a recent study performed in rural areas of Ghana, an at least 95% coverage for all three doses of DPTHH by the end of the first year of life was reported. However, immunizations suffered from poor timeliness, with substantial inequity across educational and socio-economic classes, due to weak supply chain management and poor access to health services [51]. Therefore, more detailed studies are needed to assess the efficacy of HBV vaccination in Ghana. While the administration of the hepatitis B vaccine at birth or in early childhood has been effective in reducing the incidence of the disease in many endemic regions [52, 53], immunization programs will not benefit patients already chronically infected with HBV. The low HBsAg carrier rate in the age group ≥60 years may be related to the higher mortality rate in elderly individuals with viral hepatitis [54, 55], which has been attributed in part to a higher prevalence of co-morbidities. Since HBsAg carriage was high in adolescents and adults, our data demonstrate that public health efforts are required to screen and subsequently ensure access to treatment, particularly for persons suffering from cirrhosis or advanced stages of liver disease. Moreover, additional needs for the management of concurrent or sequential infection with HBV and other viruses such as HIV, HCV and/or HDV—often associated with more severe and progressive liver disease and a higher incidence of cirrhosis and hepatocellular carcinoma—should be addressed. These include the identification and initial treatment of the dominant virus followed by monitoring of the co-infecting virus [56]. Another essential aspect to be considered in the management of hepatitis is that populations with high HBV prevalence live in regions also endemic for mycobacterial infections such as tuberculosis, leprosy and Buruli ulcer. Special caution has to be paid when treating individuals with advanced stage liver disease with standard anti-mycobacterial regimens in order to avoid drug-induced hepatitis [56–58]. In this context, drug-induced liver injury has been reported to be three- to six-fold higher in persons infected with HBV, HCV or HIV who are receiving anti-tuberculosis drugs, due to hepatotoxicity of isoniazid, rifampicin and pyrazinamide [59].

Until today only a limited number of HBV genotyping studies have been conducted in Ghana. Two reports have indicated the exclusive presence of genotype E in samples collected more than 15 years ago [34, 35]. Strains analyzed in these studies came from targeted groups, such as pregnant women, HIV seropositive individuals and blood donors. In the present study, we provide the first molecular study of HBV from carriers resident in rural communities of the Offin river valley of Ghana. Although more than a decade has passed, and the co-circulation of two genotypes, namely A and E, has been reported in other West African countries [35], our phylogenetic analysis, based on the pre-S/S sequence of 52 HBV samples, showed that all of them belonged to genotype E, and all were predicted to belong to serotype *ayw4*. However, two separate E *ayw4* genetic clusters were found. The Offin cluster, comprising 33/52 sequences obtained from individuals living in 11 of the 13 study communities, showed a low diversity with an intra-group mean distance of only 0.7%. With an intra-group mean distance of 1.6%, the 19 sequences belonging to the pan-African cluster were more diverse, and originated from nine different communities, including Akomfore, Krakrom and Mfantseman, which are the three most remote communities. The Offin river basin has been a focus of small scale gold mining for many centuries and has produced more gold than any other river system in Ghana [60]. Over the years, there has been up-scaling of mining activities coupled with the influx of artisans from other regions of Ghana, as well as of citizens from other countries. During our exhaustive household survey in the Offin river valley, we encountered a number of nationals from other West African countries, particularly from Niger, Nigeria and Benin, representing one potential source for the introduction of new HBV genetic variants into the river basin.

In general, the very low diversity of the 52 pre-S/S region (1.2%) and S region (0.6%) sequences of this study is in line with previously published data reporting a strikingly low genetic diversity of HBV/E [14, 21]. Analysis of 610 S region sequences has revealed a mean genetic diversity of 0.8% for HBV/E as compared with a 2.1% diversity for 167 African HBV/A sequences [21]. The apparently relatively recent emergence of genotype E in Africa is contrasted by the excessive spread of this genotype throughout West Africa. While this has led to hypotheses on a more efficient mechanism of transmission, as compared to genotype A, it has been speculated in a recent report that historical mass vaccination campaigns with unsafe injection needles may have caused the current high prevalence rates of HBV/E throughout sub-Saharan Africa [21]. As a result of the increasing selection pressure caused by widespread HBV vaccination, immunotherapy and chronic HBV infections, various escape mutations in the HBsAg gene have been reported. Neutralizing antibodies induced by immunization are targeted primarily against the conformational epitopes of the ‘a’ determinant of the surface antigen [47]. Mutations within this determinant may affect the binding of anti-HBs antibodies, allowing for the replication of the virus in vaccinated individuals, and pose a challenge to immunoassay detection. Although only individuals testing HBsAg positive were included in the present study, we identified the previously described [49] Ser143Leu substitution in the ‘a’ determinant. The codon 143 mutation has been reported to cause false negative results in some commercial HBsAg immuno-assays [61]. These data stress the importance of using HBsAg assays with a high sensitivity, in particular for the screening of blood bank donors. In addition, we detected two nonsense mutations in several HBV sequences at positions Leu^216^ (sLeu216*) and Trp^182^ (sTrp182*) of the S gene, for which potent oncogenic activity was recently demonstrated, suggesting a role in hepatocarcinogenesis [62]. No known resistance mutation to antiretroviral drugs was detected in our dataset, which might be explained by the lack of selection pressure due to the limited use of the drugs in this region.

In summary, we revealed a high prevalence of HBsAg carriers among adults and the occurrence of HDV co-infection in the general population of the Offin river valley of Ghana. A low genetic diversity was found among the HBV sequences, and HBV/E was the only genotype detected. We conclude that in addition to strict adherence to the EPI schedule for children, there is need for an anti-hepatitis B campaign in the form of screening and assessing the severity of liver disease in order to select individuals eligible for treatment, prioritizing patients with advanced stage liver disease. While in resource-rich countries effective treatments for chronic hepatitis are widely available, access to antiviral therapy is restricted in developing countries and should be urgently improved. Challenges associated with this endeavor have to be faced from the perspectives of global health and social justice [63].

## Supporting Information

S1 Fig Distribution of study participants by age group and residential community. (TIF)

S1 Table Origin of HBV sequences belonging to the Offin and pan-African clusters. (PDF)

S2 Table Demographic and serological data of HBsAg carriers, for which sequence information is available. (PDF)
