# Supplementary material for: Limited Genetic Diversity of Hepatitis B Virus in the General Population of the Offin River Valley in Ghana
Source: PLoS One. 2016 Jun 6;11(6):e0156864. doi: 10.1371/journal.pone.0156864 (PMC4894622; doi:10.1371/journal.pone.0156864)
Supplement: S1 Table — (PDF) [file pone.0156864.s002.pdf]

S1 Table. Origin of HBV sequences belonging to the Offin and pan-African clusters.

	**Community#**													**Total**
	ABUS	AFS	BDS	BUDS	KPS	KGS	KKS	MFS	NKS	NBUS	PKS	TNS	WMS	
Offin cluster sequences (n)	0	4	0	5	4	1	2	2	2	2	4	4	3	33
Pan-African cluster sequences (n)	0	2	1	4	2	0	3	2	0	3	1	1	0	19
**Total**	0	6	1	9	6	1	5	4	2	5	5	5	3	52

Achiase (ABUS); Akomfore (AFS); Bedomase (BDS); Dominase (BUDS); Kapro (KPS); Keniago (KGS); Krakrom (KKS); Mfantseman (MFS); Nkotumso (NKS); Ntobroso (NBUS); Pokukrom (PKS); Tontonkrom (TNS); Wromanso (WMS)
