# Supplementary material for: Limited Genetic Diversity of Hepatitis B Virus in the General Population of the Offin River Valley in Ghana
Source: PLoS One. 2016 Jun 6;11(6):e0156864. doi: 10.1371/journal.pone.0156864 (PMC4894622; doi:10.1371/journal.pone.0156864)
Supplement: S2 Table — (PDF) [file pone.0156864.s003.pdf]

S2 Table: Demographic and serological data of HBsAg carriers, for which sequence information is available.

Community/ ID	Gender	Age	Additional HBsAg carriers in household	Relevant mutations (S region)	Anti-HDV antibody positivity
**AFS068**	Female	12	NI#		No
**AFS080**	Male	18	1		No
**AFS097**	Female	33	NI	sL127P	No
**AFS101**	Female	38	NI		No
**AFS113**	Female	4	NI		No
**AFS115**	Male	27	NI	sL127P	No
**BDS103**	Male	27	1		No
**BUDS005**	Female	55	NI		No
**BUDS006**	Female	21	NI		No
**BUDS015**	Female	13	NI		No
**BUDS023**	Male	13	NI		No
**BUDS033**	Female	23	NI		No
**BUDS038**	Male	8	NI		No
**BUDS051**	Female	17	NI		No
**BUDS073**	Female	35	1 (BUDS081)	sL216*	Yes
**BUDS081**	Male	21	1 (BUDS073)		No
**KGS053**	Female	32	NI		No
**KKS011**	Male	17	NI		No
**KKS013**	Male	18	2 (KKS062, KKS064)		No
**KKS033**	Male	15	NI		No
**KKS062**	Female	10	2 (KKS013, KKS064)		No
**KKS064**	Male	15	2 (KKS013, KKS062)	sL127I	No
**KPS021**	Male	4	NI		No
**KPS029**	Male	24	NI		No
**KPS078**	Male	8	1		No
**KPS088**	Female	19	NI		No
**KPS091**	Male	36	1 (KPS094)		No
**KPS094**	Male	13	1 (KPS091)		No
**MFS014**	Female	31	NI		No
**MFS022**	Female	5	NI		No
**MFS043**	Male	19	NI		No
**MFS048**	Female	25	NI	sS143L	No
**NBUS058**	Female	42	NI		No
**NBUS062**	Female	42	1 (NBUS070)		No
**NBUS067**	Male	45	NI		No
**NBUS070**	Female	23	1 (NBUS062)	sL127P	No
**NBUS078**	Male	29	NI		No
**NKS010**	Female	42	1 (NKS020)		No

**NKS076**	Male	34	NI		Yes
**PKS003**	Male	27	NI		No
**PKS034**	Male	9	2	sL216*	No
**PKS066**	Female	13	NI		No
**PKS077**	Female	35	NI		No
**PKS096**	Female	17	NI		No
**TNS014**	Female	50	1	sS140L, sL216*	No
**TNS055**	Male	28	NI	sL216*	No
**TNS065**	Female	55	NI	sL216*	No
**TNS088**	Male	14	2 (TNS091)		Yes
**TNS091**	Male	16	2 (TNS088)	sS143L	No
**WMS009**	Female	23	2 (WMS008)	sL216*	No
**WMS081**	Male	25	NI		No
**WMS082**	Male	12	NI		No

NI = not identified
